# STAT3 and mTOR: co-operating to drive HIF and angiogenesis

**DOI:** 10.18632/oncoscience.272

**Published:** 2015-11-23

**Authors:** Kayleigh M. Dodd, Andrew R. Tee

**Affiliations:** Institute of Cancer and Genetics, Cardiff University, Cancer and Genetics Building, Heath Park, Cardiff, UK

**Keywords:** mTOR, STAT3, HIF, angiogenesis, VEGF

Our understanding of angiogenic signalling has been significantly enhanced through studies of a rare genetic disorder called Tuberous Sclerosis Complex (TSC). TSC patients are predisposed to highly vascularised tumours, where renal angiomyolipomas produce high levels of vascular endothelial growth factor (VEGF) that can be readily detected. It is well established that VEGF is driven through hypoxic signalling, with the transcription factor hypoxia inducible factor-1α (HIF-1α) playing a crucial role in its expression. Early studies using cell line models of TSC uncovered that the mammalian target of rapamycin complex 1 (mTORC1) is a key mediator of HIF-1α synthesis, and highlighted the anti-angiogenic properties of mTORC1 inhibitors [1]. Herein we review our recent findings characterising mTORC1 mediated regulation of HIF-1α and discuss the clinical implications of our work.

We demonstrated that mTORC1 drives HIF-1α expression via three mechanisms, promoting not only the transcription of HIF-1α mRNA via signal transducer and activator of transcription 3 (STAT3), but also its translation via both eukaryotic initiation factor 4E-binding protein 1 (4E-BP1) and ribosomal protein S6 kinase 1 (S6K1). This drive in HIF-1α activity downstream of mTORC1 explains why the tumours which present in TSC are so heavily vascularised, and accounts for the anti-tumorigenic properties of mTOR inhibitors used in this setting. In concordance with this, we observe a 10 fold-increase in HIF-1α transcriptional activity under hypoxia with TSC2 loss, highlighting the significant impact mTORC1 activation can have on HIF-1α.

Whilst mTORC1 can promote the transcriptional activity of STAT3 through direct phosphorylation of Ser727, STAT3 is also subject to regulation from a number of different cytokines and growth factors which signal through the receptor tyrosine kinase JAK2 [2]. Both JAK2/STAT3 and mTORC1 signalling pathways are frequently activated in a wide range of malignancies and converge at the level of HIF-1α (see Figure 1). Whilst mTOR inhibitors are effective at blocking Ser727 phosphorylation of STAT3, we were able to completely abolish HIF-1α expression by targeting both the JAK2-mediated Tyr705 phosphorylation site and the mTORC1-mediated Ser727 site. Our work indicates that targeting STAT3 in parallel to mTORC1 could enhance the anti-angiogenic and anti-tumorigenic properties of mTOR inhibitors that are currently in clinical use [3].

**Figure 1:**
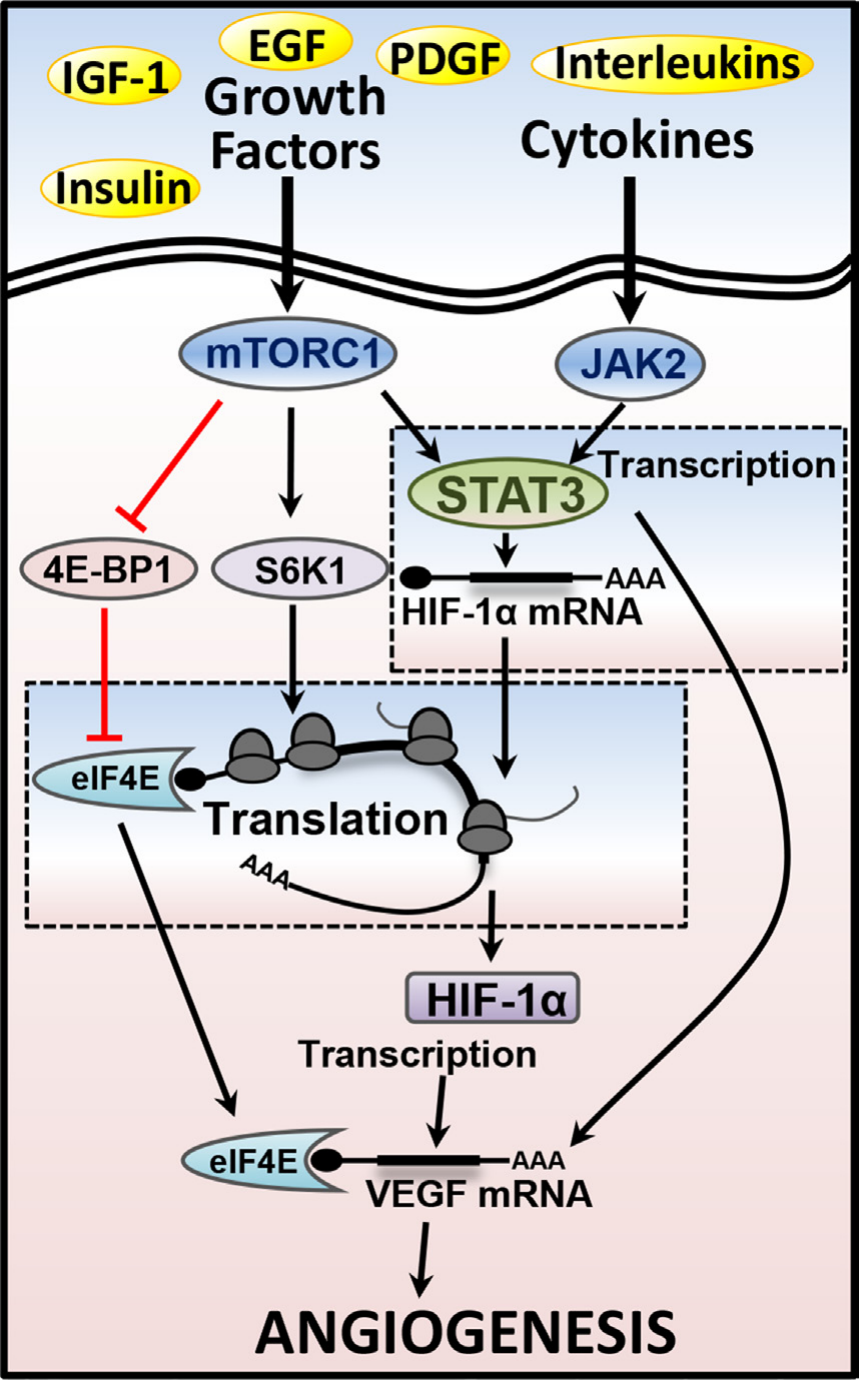
Multifaceted regulation of HIF-1α/VEGF-A via mTORC1 and STAT3 Through RTKs, growth factors (IGF-1, Insulin, and PDGF) and cytokines (Interleukins) activate mTORC1 and STAT3, which are both upstream of HIF-1α. Downstream from both mTORC1 and JAK2, STAT3 drives HIF-1α mRNA gene-expression. mTORC1 through repression of the translation inhibitor, 4E-BP1, leads to enhanced translation of HIF-1α via eIF4E. S6K1 further enhances the translation rates of HIF-1α. Both STAT3 and HIF-1α drives angiogenesis through gene-expression of VEGF-A, while mTORC1 enhances VEGF-A translation via eIF4E.

Growth of tumours in renal cell carcinoma (RCC) is highly dependent on mTORC1, HIF and VEGF which drive a pro-angiogenic response. In the microenvironment of the kidney, angiogenic signalling is crucial for metabolic transformation and malignancy. Although there has been much investment into drug discovery and the development of inhibitors that directly inhibit HIF, none of these compounds are currently suitable for clinical use. Consequently, current angiogenic therapies have been mainly restricted to inhibition of mTORC1 and the VEGF-receptor (VEGFR). Given that our work now positions STAT3 as an immediate upstream regulator of HIF, drug targeting of STAT3 may be an alternative therapeutic strategy for the treatment of vascularized tumours.

We also identified STAT3 as a common point of convergence for many receptor tyrosine kinases involved in the angiogenic response within Neurofibromatosis 1 (NF1) associated malignant peripheral nerve sheath tumours (MPNSTs) [4], reflecting the wider implications of this work. We uncovered that STAT3 inhibition or shRNA knockdown was sufficient to completely ablate expression of HIF-1α, HIF-2α and VEGF-A. Previous studies indicated that rapamycin repressed the growth of NF1-associated malignancies, but with minimal effects upon HIF-1α expression indicating that STAT3 primarily drives HIF-1α activity in NF1 [5]. STAT3 is considered an oncogene and mediates a range of cellular processes associated with tumourigenesis, increasing its appeal as a potential therapeutic target [2]. In keeping with this, knockdown of STAT3 inhibited cell migration and blocked tumour spheroid formation in a range of different MPNST cell lines tested [4].

A current limitation of anti-angiogenic therapies targeting the VEGF receptor (VEGFR) is acquired drug resistance. This occurs through activation of compensatory signalling pathways coordinating tumour revascularisation, where a multitude of pro-angiogenic factors (such as VEGF, FGFs, EGF, and IL-8 (as well as others)) signal through a network of target receptor tyrosine kinases (RTK) to elicit the pro-angiogenic response. Consequently, angiogenic signalling is highly flexible and driven through cross-talk between multiple RTKs. This level of flexibility hampers current tyrosine kinase inhibitor therapies [6]. Given that STAT3 is a common downstream target of multiple RTKs, acquired resistance may be less of a problem in this setting. STAT3 is also fairly indispensable in normal cellular function, suggesting that the side effect profile of STAT3 inhibitors should be low. Collectively, our work highlights the need for the development of clinically available STAT3 inhibitors for cancer therapy.

## CONCLUSIONS

mTORC1 inhibitors such as everolimus are clinically approved for the treatment of tuberous sclerosis, breast cancer and renal cell carcinoma. Our work indicates targeting STAT3 alongside mTORC1 could enhance the anti-angiogenic efficacy of everolimus, blocking HIF-1α expression at the level of both transcription and translation.
